# A micro-vibration-driven direct ink write printing method of gallium–indium alloys

**DOI:** 10.1038/s41598-023-31091-z

**Published:** 2023-03-08

**Authors:** Sheng Lin, Long Zhang, Liang Cong

**Affiliations:** School of Mechanical Engineering, Dalian Jiaotong University, Dalian, 116028 People’s Republic of China

**Keywords:** Mechanical engineering, Metals and alloys, Fluids

## Abstract

Combining liquid fluidity and metallic conductivity, gallium–indium (Ga–In) alloys are making a splash in areas such as stretchable electronic circuits and wearable medical devices. Due to high flexibility, direct ink write printing is already widely employed for printing Ga–In alloys. Currently, pneumatic extrusion is the main method of direct ink write printing, but the oxide skin and low viscosity of the Ga–In alloys make it challenging to control after extrusion. This work proposed a method for direct ink write printing of Ga–In alloys utilizing micro-vibration-driven extrusion. Micro-vibration reduces the surface tension of Ga–In alloy droplets and avoids the appearance of random droplets during printing. Under micro-vibration, the nozzle tip pierces the oxide skin to form small droplets which have a high moldability. The droplet growth process is significantly slowed down by optimizing suitable micro-vibration parameters. Therefore, the Ga–In alloy droplets with high moldability can be maintained at the nozzle for a long period, which improves printability. Furthermore, better printing outcomes were obtained with micro-vibrations by choosing the proper nozzle height and printing speed. Experiment results demonstrated the superiority of the method in terms of Ga–In alloys extrusion control. With this method, the printability of the liquid metals is enhanced.

## Introduction

Gallium-based alloys, which are liquid metals with low melting points, are commonly used in flexible electronics^1,2^, materials synthesis^3,4^, stretchable electronics^5,6^, sensors^7,8^, and other areas due to their unique physical properties. The molding capabilities of Ga–In alloys are improved by combining 3D printing technology. However, Ga–In alloys are oxidized rapidly in the air to form a natural oxide skin which is a viscoelastic material^9^. The oxide skin dominates the rheological properties and reduces the surface tension^10^, which is the key to achieving Ga–In alloys printing^11^. In the pneumatic extrusion Ga–In alloys 3D printing process, the oxide skin causes large Ga–In alloy droplets at the nozzle, decreasing moldability^12^. Then, the extrusion process is difficult to control. The difficulty of controlling extrusion makes Ga–In alloys generate random-sized droplets during the printing process. These random-sized droplets will affect the requirements of resolution^13,14^ and conductivity^15^ of the printed structure. Hence, many researchers have proposed methods to avoid the creation of random-sized droplets. Three methods are adopted to assist the pneumatic extrusion of liquid metals.

The printing is realized by breaking the oxide skin through an external force. Cook et al.^16^ proposed that the droplets were extruded but not dropped by precisely controlling the extrusion pressure, and the shear force between the droplets and the substrate was used to adhere the liquid metals to the substrate. Ladd et al.^17^ ruptured the oxide skin by tensile force to form free-standing liquid metal wires. However, the method of destroying the oxide skin by external force usually puts forward higher requirements for the printing process, such as precise control of nozzle height. The rheological characteristics of liquid metals were changed by combining metallic or nonmetallic materials, enabling the liquid metal to keep its shape even after extrusion. Wu et al.^14^ proposed a liquid microgel ink by mixing sodium alginate microgel, which reduced the enormous surface tension and enhanced the adhesion performance. Chan et al.^13^ suggested a recyclable and reversible liquid metal paste by combining SiO_2_ particles, which improved the adhesion properties of liquid metals. According to Daalkhaijav et al.^18^ adding conductive nano- or micro-nickel materials to liquid metals would improve their elastic modulus and yield stress, and enable 3D printing. The problem of precise control of extruded liquid metal can be effectively solved by adding other materials, but the application is also limited by the added materials. Coaxial co-extrusion printing was reached by redesigning the nozzle’s mechanical structure. To obtain a continuous and stable liquid metal flow, Khondoke et al.^19^ developed a coaxial co-extrusion nozzle that could wrap liquid metal in a thermoplastic elastomer and extrude it together. Wu et al.^20^ suggested a coaxial nozzle with the inner nozzle extension to wrap and extrude liquid metal steadily and effectively, which could acquire multi-resolution liquid metal printing. But the 3D structure cannot be printed by stacking liquid metal droplets with this method. The above method partially solves the problem of oxide skin in the process of liquid metal printing, but the printing process, materials or liquid metal formed structures are limited to some extent. In order to reduce the influence of oxide skin on printing results without limiting the material or process, we proposed a micro-vibration driving 3D printing method for liquid metal extrusion. Using this method, oxide skin of the droplet is broken when the droplet does not expand to a sufficient size. This method will effectively avoid the occurrence of random droplets in the printing structure.

Here, a micro-vibration-driven method of Ga–In alloys extrusion is proposed to attain the printing. The surface tension was reduced under vibration according to the measurement of the surface tension of the Ga–In alloy droplets. The moldability of Ga–In alloy droplets was increased under vibration. The extrusion speed of droplets can be controlled to the desired value by selecting suitable vibration parameters. Thus, droplets with high moldability can be maintained in the nozzle for desired time. The random large droplets in the printing track are avoided without the limitation of materials and the printing process.

## Materials and method

### General information

Eutectic Gallium–indium (EGaIn) (%75 Ga, %25 In) was chosen as the liquid metal material. Gallium and indium were weighed in a 3:1 ratio at a concentration of 99.99% each. Gallium was purchased from East Hope Group Co., Ltd. Indium was purchased from Zhuzhou Smelting Group Co., Ltd. The physical properties of the EGaIn are shown in Table 1. The properties of the EGaIn droplets extruded under micro-vibration directly determine the printing quality. Thus, the experimental research on the surface tension, moldability, droplet residence time, and extrusion speed of the droplets was carried out. The EGaIn printing experiments under micro-vibration were also conducted to analyse the influence of nozzle height and printing speed on the printing results. Finally, a flexible sensor was made by using micro-vibration extrusion liquid metal 3D printing method.

**Table 1:** The physical properties of EGaIn.

	EGaIn
Density (g cm^−3^)	6.25
Melting point (°C)	15.5
Viscosity (mPa s)	1.99
Surface tension (mN/m)	624
Electrical conductivity (10^7^ m^−1^)	3.4

### Principle of micro-vibration piercing oxide skin

**Figure 1:**
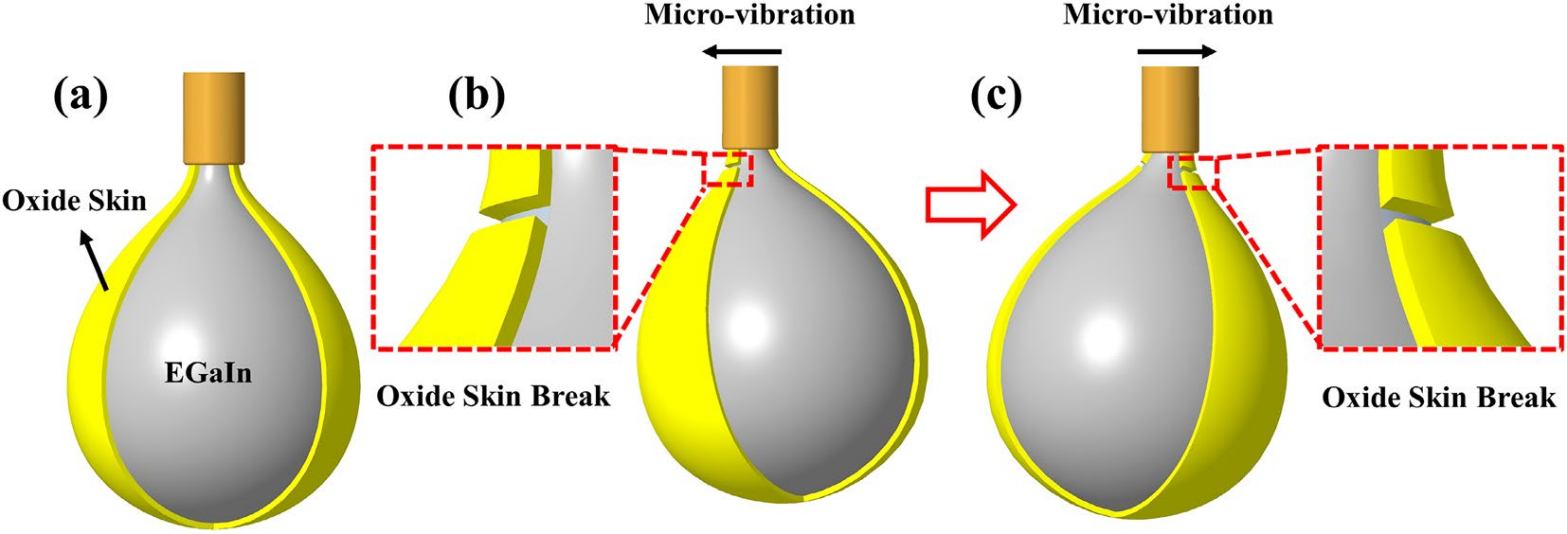
Principle of micro-vibration piercing oxide skin. (**a**) EGaIn forms a core–shell structure in air. (**b**, **c**) Micro-vibrations cause the oxide skin to break.

As shown in Fig. 1a, EGaIn is extruded from the nozzle and is rapidly oxidized to form a core–shell structure in air. The oxide skin causes EGaIn to form a drop at nozzle. Figure 1b and c show the destroying process of the oxide skin with micro-vibration driven method. Under the vibration force, the nozzle pieces the nearby oxide skin, making the droplet drop ahead of time when the oxide skin does not reach the yield stress limit. Thus, the desired extrusion speed and moldability can be obtained by selecting suitable vibration parameters.

### Experimental system construction

**Figure 2:**
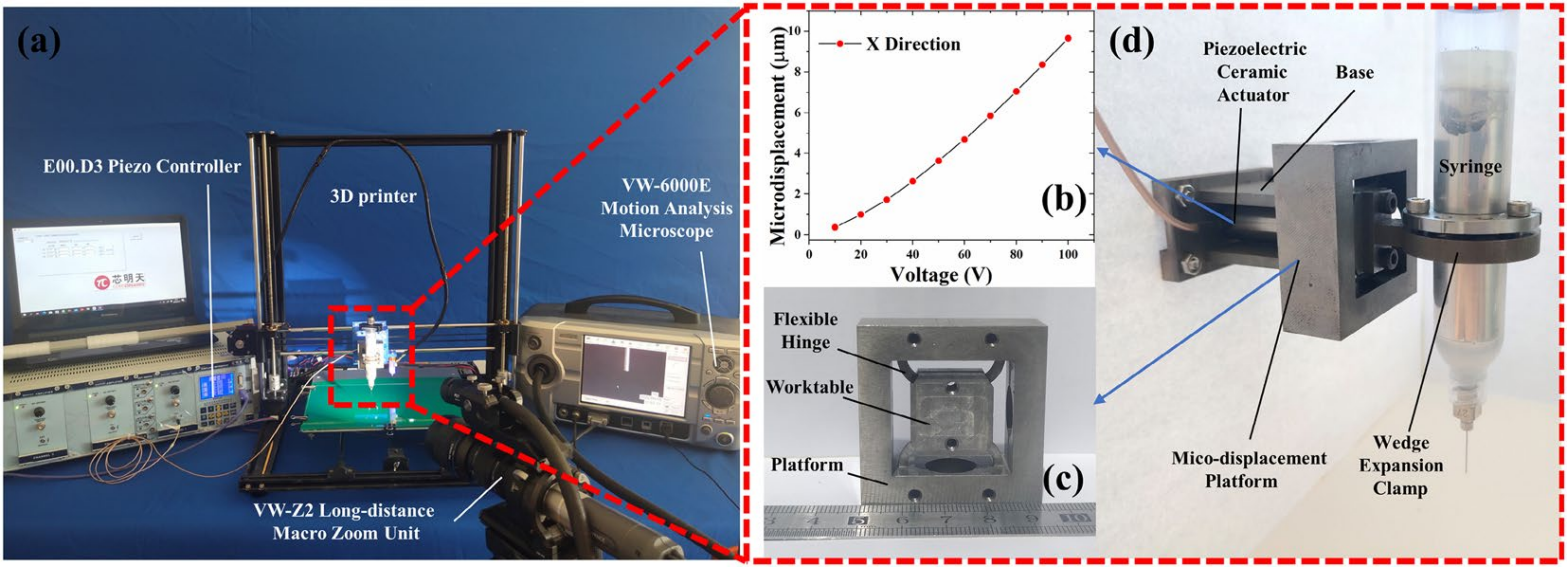
Experimental equipment. (**a**) Composition of experimental system. (**b**) The relationship between voltage and output displacement. (**c**) Micro-displacement platform. (**d**) Micro-vibration mechanism.

The experimental equipment consists of a micro-vibration mechanism, a high-speed camera, and a 3D printer, as shown in Fig. 2a. The status of extruded EGaIn was recorded by a high-speed camera. The high-speed camera includes the VW-6000E motion analysis microscope and the VW-Z2 long-distance macro zoom unit from KEYENCE. An Anet A8-Plus 3D printer was chosen as motion control mechanism. The printing material feeding system of the original 3D printer was removed and the micro-vibration mechanism was assembled on the position. Figure 2d shows the micro-vibration mechanism, which is composed of micro-displacement platform, piezoelectric ceramic actuator and wedge expansion clamp. The micro-displacement platform is shown as Fig. 2c. Piezoelectric ceramic actuator was fixed on the platform to drive the worktable. The E00.D3 Piezo Controller was used to control the low voltage columnar preloaded piezo actuators (PSt150/7/20) from Harbin Core Tomorrow Technology Co., Ltd. in China. Wedge expansion clamp was fixed on the worktable, and the syringe was clamped on the wedge expansion clamp. The nozzle on the syringe is 0.5 inches long and 0.2 mm inner diameter. The base connected the micro-vibration mechanism and the 3D printer. Figure 2b shows the relationship between the voltage of piezoelectric ceramics and the output displacement of the compliant mechanism worktable. The DC drive voltage increased from 10 to 100 V at an interval of 10 V. The displacement of the compliant mechanism worktable was measured by the LVDT micrometer of Harbin Core Tomorrow. In the micro-vibration extrusion 3D printing for EGaIn, the sine excitation signal $V={V}_{0}\mathrm{sin}\left(2\uppi ft\right)$ was applied to the piezoelectric ceramic driver, where, ${V}_{0}$ is the amplitude of the voltage, $f$ is the frequency. The output displacement of the worktable is $X={X}_{0}\mathrm{sin}\left(2\uppi ft+\varphi \right)$, where, ${X}_{0}$ is the amplitude, $f$ is the vibration frequency, and $\varphi$ is the phase angle. The curve in Fig. 2b proposed the relationship between ${X}_{0}$ and ${V}_{0}$.

### Experiments on the properties of EGaIn droplets extruded with micro-vibration

#### Experiments on surface tension of the EGaIn droplets

An EGaIn droplet suspends in the air for the balance of gravitation and surface tension. The dimensionless ratio of the gravitational and surface tension forces is defined by the relation^21^

1 $$Bo=\frac{\Delta \rho \mathrm{g}{R}^{2}}{\sigma }$$

where, $Bo$ denotes the Bond number, $\Delta \rho$ is the density difference between the liquid and the surrounding fluid, $\mathrm{g}$ is the Earth’s gravitational constant, $\sigma$ is the surface tension, and *R* is the radius of curvature at the drop apex.

In order to clearly express the surface tension, Eq. (1) can be rewritten as

2 $$\sigma =\frac{\Delta \rho \mathrm{g}{R}^{2}}{Bo}$$

In Eq. (2), $\Delta \rho$ and $\mathrm{g}$ are known. If the Bond number $Bo$ and the drop radius *R* at the apex are determined, the surface tension $\sigma$ can be obtained.

**Figure 3:**
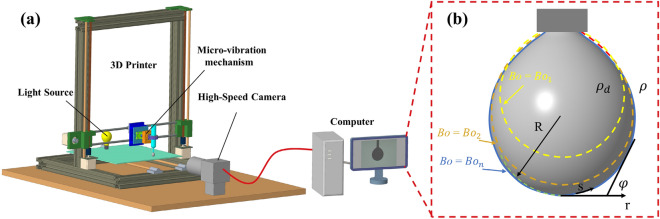
Acquisition process of EGaIn droplet property (**a**) EGaIn droplet image extraction. (**b**) Analysis of EGaIn droplet property.

$Bo$ and *R* can be determined by matching the measured drop profile to a theoretical drop contour calculated according to the Young–Laplace equation in open-source software OpenDrop^22^. The process is shown in Fig. 3. In Fig. 3a, The high-speed camera records change of the droplets at a frame rate of 60 fps. The experimental images from high-speed camera are loaded in software OpenDrop. The EGaIn density, air density and nozzle outer diameter are imported in the OpenDrop and droplet profiles is extracted. $Bo$ and *R* are obtained by minimising the sum of squared residuals of the theoretical pendant drop profile and experimental data as shown in Fig. 3b. And the surface tension $\sigma$ is calculated by Eq. (2).

#### Experiments on moldability of the EGaIn droplets

EGaIn’s ability to maintain a stable microstructure within its oxide skin at room temperature is named as moldability^12^. The surface area of the droplet is denoted as *A*, and the volume of the droplet is denoted as *V*. The moldability increases as the *A*/*V* ratios^23^. Therefore, the change law of moldability can be reflected by the change law of *A*/*V*.

The fitted Young–Laplace solution can also be used to give additional data, such as volume *V* and surface area *A*^24^

3 $$V=\uppi \int {\overline{r} }^{2}\mathrm{sin}\varphi \mathrm{d}\overline{s }, A= 2\uppi \int \overline{r}\mathrm{d }\overline{s }$$

4 $$A/V=\boldsymbol{ }\frac{2\int \overline{r}\mathrm{d }\overline{s} }{\int {\overline{r} }^{2}\mathrm{sin}\varphi \mathrm{d}\overline{s} }$$

where, the bar indicates dimensionless quantities, $r$ is column coordinates, $\varphi$ is tangent angle, $s$ is arc length. To compare the moldability of the droplets, 25 images are intercepted as the droplet grew larger and calculated the moldability. The volume *V* and surface area *A* of the droplet are obtained by Eq. (3). The ratio of *A*/*V* is obtained from Eq. (4).

#### Experiments on the average extrusion speed of the EGaIn droplets

The extrusion speed of the EGaIn also has an impact on the printing results. When the extrusion speed is low, the EGaIn is not sufficiently extruded, resulting in the printed structure being stretched or fractured. When the extrusion speed is high, the printing traces can exhibit bulges or form droplets. So, it is necessary to investigate the effect of micro-vibrations on the extrusion speed of EGaIn. The average extrusion speed can be calculated by Eq. (5),

5 $$\overline{v }=\frac{w}{t}$$

where, $\overline{v }$ is average extrusion speed, *t* is extrusion time, *w* is the weight of extruded EGaIn in *t* time.

The specific experimental process is as follows,

1. The range of voltage amplitude is from 10 to 100 V at 10 V intervals. The voltage amplitude is initially selected as 10 V, repeat step (2) for different voltage amplitudes.
2. The vibration frequency is selected from 100 to 600 Hz at 100 Hz intervals, repeat steps (3)–(8) for different vibration frequencies.
3. The syringe is initially filled with 50 g of EGaIn.
4. Pre-extrusion is carried out under micro-vibration until the EGaIn can be extruded smoothly.
5. The EGaIn extrusion process is recorded by a high-speed camera. The extrusion process lasts for 20 s, and the weight of the extruded EGaIn is measured.
6. The average extrusion speed can be calculated by Eq. (5).
7. After 2 g of EGaln has been extruded, repeat steps (5)–(7).
8. The EGaIn in the syringe gradually decreases until the EGaIn cannot be extruded. The experimental is finished.

### Experiments on printing parameter influence

In addition to the EGaIn droplet property affecting the printing result, printing speed and nozzle height also have an important impact on the printing result. Select 80 V and 400 Hz as vibration parameters according to the experiment results of droplet properties. The nozzle height from the substrate gradually increased from 0.03 to 0.08 mm, and the printing quality was observed. Select the appropriate nozzle height 0.05 mm, adjust the printing speed from 0.5 to 2.5 mm/s, and analyze the printing results.

### Micro-vibration printing application

**Figure 4:**
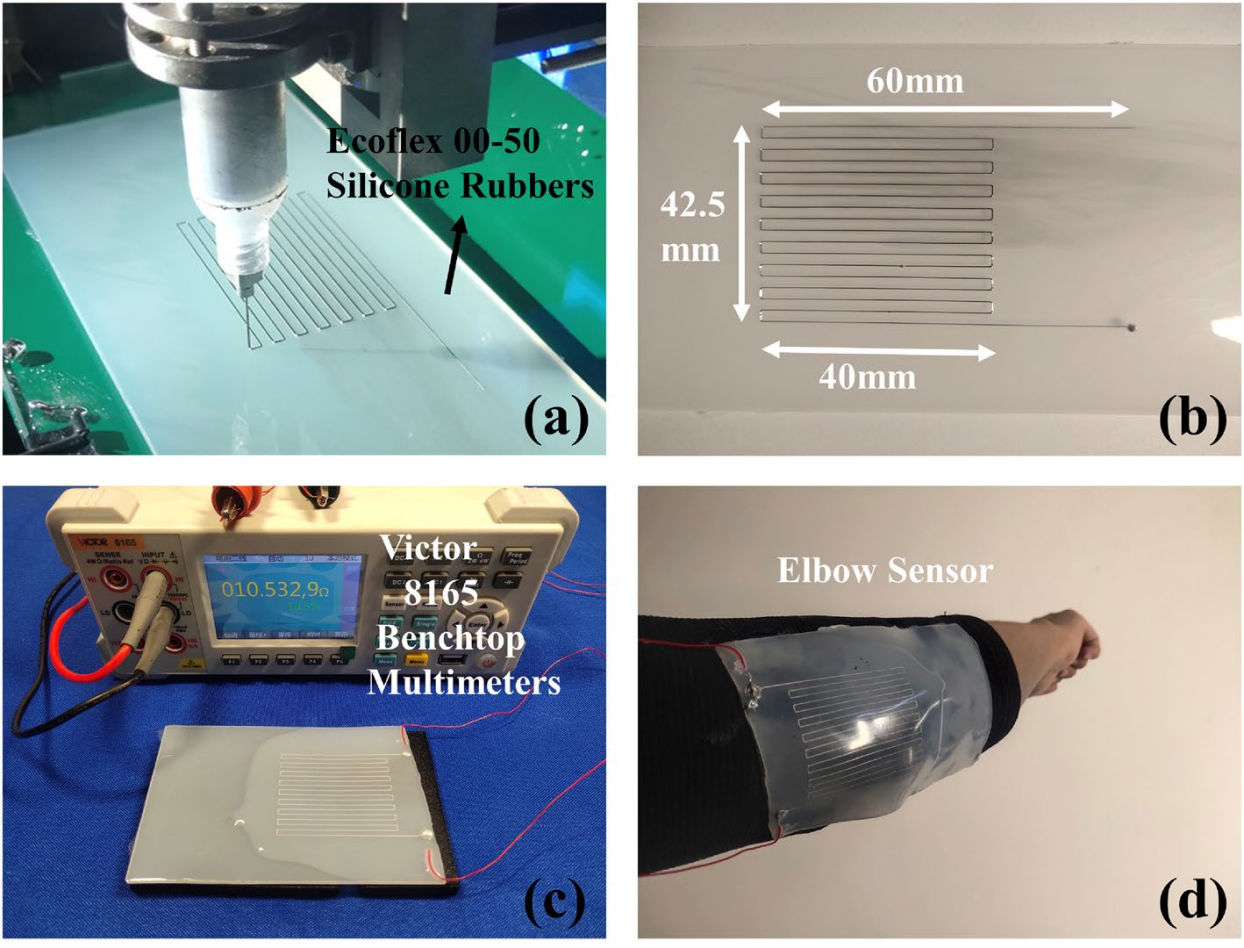
Micro-vibration printing application. (**a**) Printing process for sensor structure. (**b**) Size of the sensor structure. (**c**) Sensor conductivity display (**d**) Wearing of the elbow sensor.

Based on the micro-vibration-driven printing method, the elbow flexible sensor was created. The printing process is shown in Fig. 4a. The base material of the flexible sensor is Ecoflex silica gel. The construction and dimensions of the sensor are shown in Fig. 4b. After 3D printing, the EGaIn track was packaged with silica gel, as shown in Fig. 4c. And the display of high-precision multimeter shows that the sensor has good conductivity. The wearing is shown in Fig. 4d.

## Results and discussion

### Influence of micro-vibration parameters on surface tension and moldability

**Figure 5:**
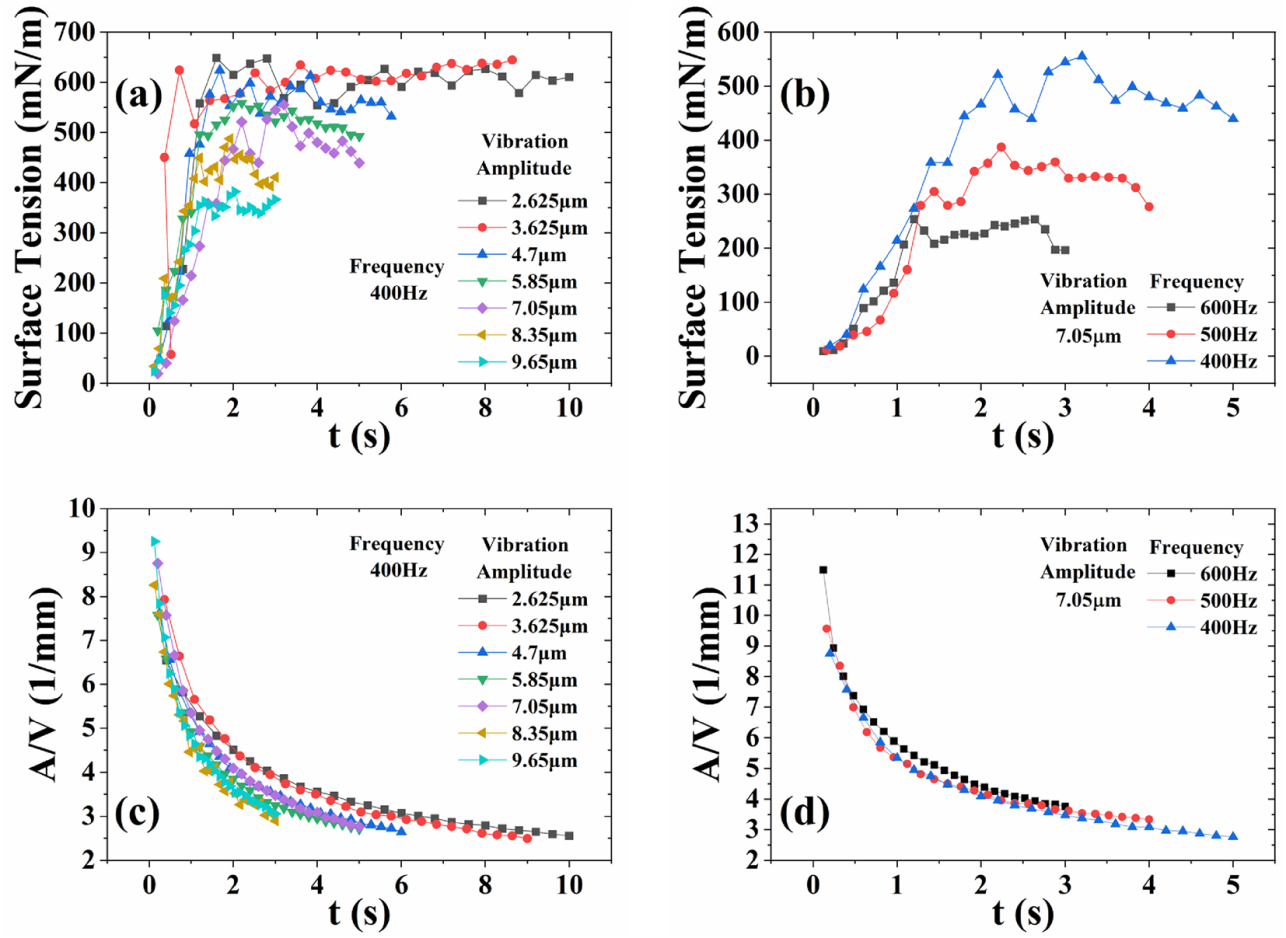
Surface tension and Moldability. (**a**) Influence of vibration amplitude on the surface tension of droplets. (**b**) Influence of vibration frequency on the surface tension of droplets. (**c**) Influence of vibration amplitude on the moldability of droplets. (**d**) Influence of vibration frequency on the moldability of droplets. In the experiment, the weight of the EGaIn in the syringe is 50 g.

Each curve in Fig. 5a and b represents the trend of the surface tension of the EGaIn droplets during the extrusion of individual droplet. Figure 5a shows that the extrusion time of a single droplet gradually decreases as the vibration amplitude increases. And at different vibration amplitudes, the surface tension of the droplets tends to stabilize. The surface tension of the droplets gradually reduces as the amplitude of vibration increases. When the vibration amplitude is small, such as 2.625 µm and 3.625 µm, the effect on surface tension is not obvious. The droplets still have a high surface tension of about 600 mN/m. The higher surface tension causes EGaIn to form droplets during the printing process, which affects the printing quality. When the vibration amplitude is large, the surface tension of the droplets decreases significantly. When the vibration amplitude is 9.65 µm, the surface tension of the droplets is about 350 mN/m. Thus, the nozzle tip can pierce the oxide skin easier when the vibration amplitude is larger. Figure 5b indicates that the extrusion time of individual droplet decreases as the vibration frequency increases. This means that the increase of vibration frequency can help the nozzle tip pierce the oxide skin. The surface tension of the droplets gradually decreases as the vibration frequency increases. The surface tension at 500 Hz and 600 Hz vibration frequency is about 330 mN/m and 240 mN/m.

Moldability is discussed by discussing the change rule of *A*/*V* because the change trend of moldability is consistent with that of *A*/*V*. Figure 5c shows that the moldability of the droplet overall increases as the vibration amplitude increases during the process of the individual droplet becoming larger. When the vibration amplitude is 2.625 µm, 4.7 µm and 9.6 µm, the *A*/*V* varies in the ranges of [2.56–6.73], [2.64–7.84] and [3.06–9.25], respectively. Therefore, the EGaIn has higher moldability at higher vibration amplitudes. But the individual droplet extrusion time is short at higher vibration amplitude. A suitable vibration amplitude needs to be chosen so that droplets with high moldability have a long residence time at the nozzle tip. Hence, a vibration amplitude of 7.05 µm was chosen for the subsequent printing experiments. The input sine voltage amplitude corresponding to the amplitude of 7.05 µm is 80 V. Figure 5d shows that as the vibration frequency increases, the droplet’s moldability increases overall. The variation ranges of *A*/*V* are [2.77–8.76], [3.34–9.57] and [3.77–11.5] for 400 Hz, 500 Hz and 600 Hz vibration frequencies, respectively. As the vibration frequency increases, the extrusion time of individual droplets decreases. The right vibration frequency needs to be selected to combine the high moldability with the long residence time. The *A*/*V* of the droplets are very similar at 400 Hz and 500 Hz frequencies. However, the droplet has a significantly longer residence time at 400 Hz. Hence, a vibration frequency of 400 Hz was chosen for printing experiments.

### Comparison of droplets properties under pneumatic driving and micro-vibration driving

**Figure 6:**
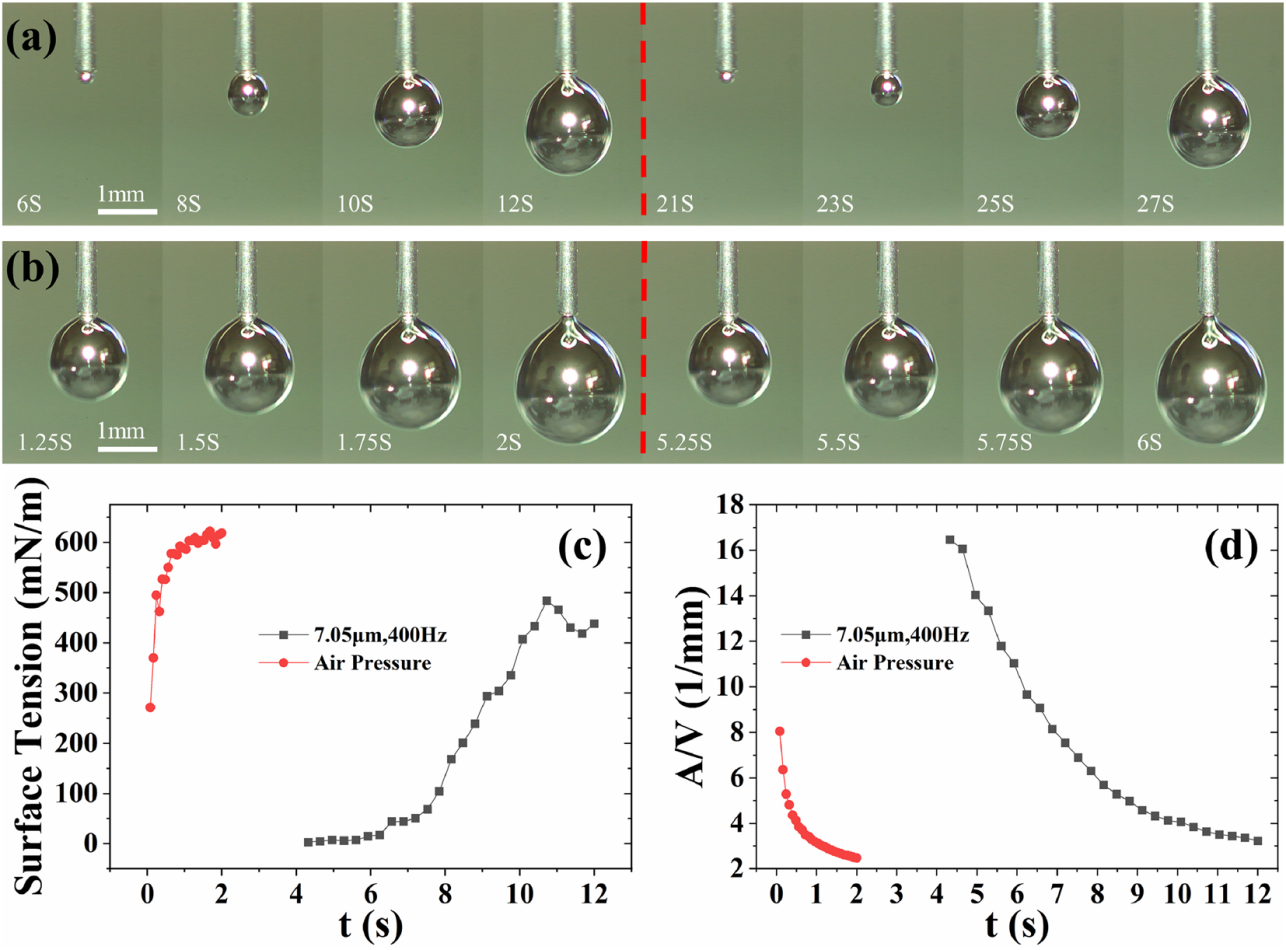
Comparison of micro-vibration and pneumatic. (**a**) The EGaIn extrusion process under micro-vibration. (**b**) The EGaIn extrusion process under pneumatic. (**c**) Surface tension of droplets extrusion under micro-vibration and air pressure. (**d**) Moldability of droplets extrusion under micro-vibration and air pressure. In the experiment, the weight of the EGaIn in the syringe is 24 g.

Figure 6a shows the extrusion process of the droplets under micro-vibration. The vibration parameters are 7.05 µm vibration amplitude and 400 Hz vibration frequency. Figure 6b is the extrusion process of EGaIn with air pressure. The pressure was controlled using a pressure-reducing valve and EGaIn can be extruded at a minimum air pressure of 11 kPa. In 20 s, 2.732 g of EGaIn was extruded under air pressure and 0.08 g of EGaIn was extruded under vibration. The average extrusion speed under air pressure and vibration can be calculated. The pneumatic drive was 34.15 times faster than micro-vibration. The extrusion times of individual droplet under micro-vibration and pneumatic pressure are 8 s and 2 s, respectively. Figure 6c indicates that the droplets have a high surface tension under air pressure, about 600mN/m. However, the overall surface tension is relatively low under vibration, and the surface tension is about 430mN/m when the droplet reaches the maximum size. Figure 6d shows that the various ranges of *A*/*V* under micro-vibration and air pressure are [2.48–8.05] and [3.23–16.48], respectively. Furthermore, The moldability of pneumatic driving decreases faster than that of micro-vibration drive. Such as, the *A*/*V* of the droplet rapidly decreases to 3.85 1/mm after 0.5 s of extrusion under air pressure. Under vibration, the A/V decreases to 3.85 1/mm in the 10th s, i.e., after 6 s of extrusion. This indicates that micro-vibration significantly reduces the extrusion speed of EGaIn, allowing the droplets with high moldability to stay for a long time at the nozzle tip, which enhances the printability of EGaIn.

#### Influence of micro-vibration parameters, weight and nozzle inner diameter on average extrusion speed

**Figure 7:**
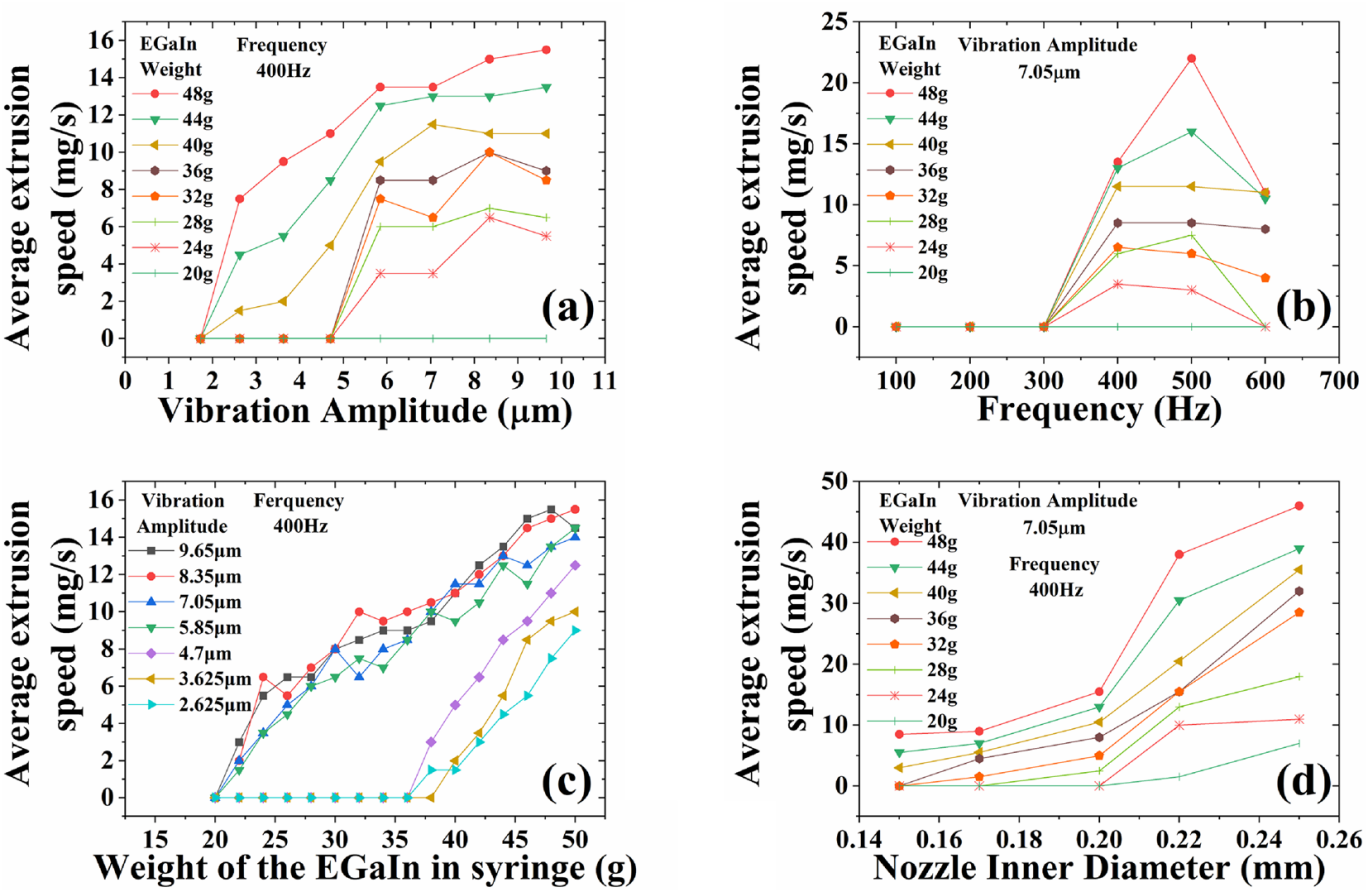
Influence of vibration parameters, weight and nozzle inner diameter on extrusion speed. (**a**) Influence of vibration amplitude on the average extrusion speed. (**b**) Influence of vibration frequency on the average extrusion speed. (**c**) Influence of the weight of the EGaIn in the syringe on the average extrusion speed. (**d**) Influence of different nozzle inner diameters on the average extrusion speed.

Figure 7a indicates that the average extrusion speed of EGaIn increases as the vibration amplitude increases. Figure 7b demonstrates that the average extrusion speed of the EGaIn increases and then decreases as the vibration frequency increases, reaching a peak at a certain frequency. Figure 7c shows that the average extrusion speed of EGaIn increases with the increment of the EGaIn weight in the syringe. The above micro-vibration extrusion experiments were carried out with a 0.2 mm inner diameter nozzle. In order to widen the range of nozzles available for printing, the influence of different inner diameter nozzles on the average extrusion speed of EGaIn was measured. As shown in Fig. 7d, the average extrusion speed of EGaIn increases as the nozzle inner diameter increases.

#### Influence of printing parameters on printing structure under vibration

**Figure 8:**
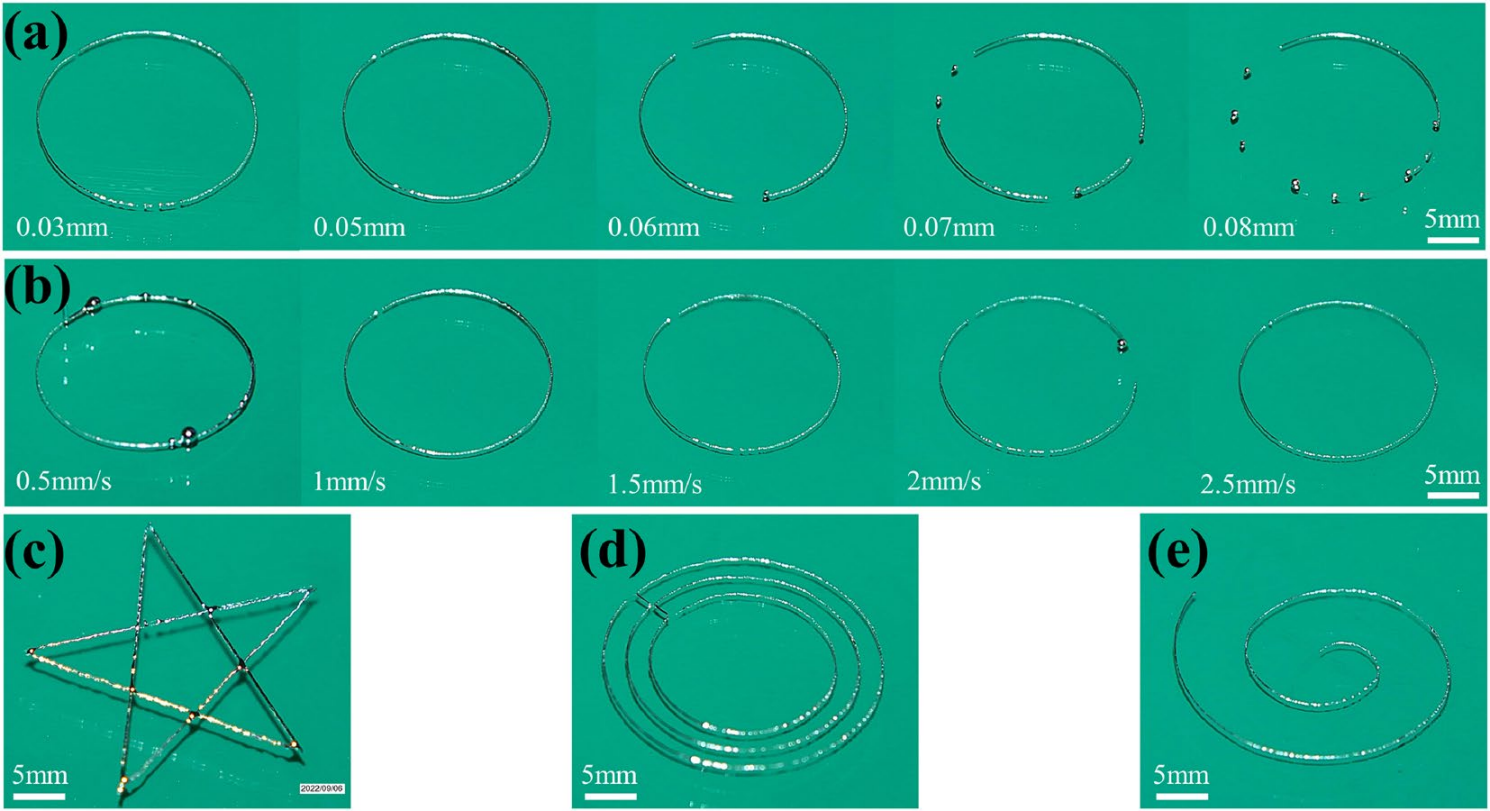
3D printing experiment of EGaIn driven by micro-vibration. (**a**) Printing track using various nozzle heights, printing speed: 1 mm/s. Vibration variable: 80 V, 400 Hz, 24 g. (**b**) Printing track using various printing speeds, nozzle height: 0.05 mm. Vibration variable: 80 V, 400 Hz, 24 g. (**c**) Five-pointed star. (**d**) Concentric circle. (**e**) Spiral line.

According to Fig. 8a, the extruded EGaIn generated droplets on the substrate when the nozzle height was higher than 0.05 mm. When the nozzle height was too lower, the printed EGaIn track was thinner because droplets didn’t reach enough size. The effect of printing speed on printing is shown in Fig. 8b. When the printing speed was below 1 mm/s, droplets formed on the substrate because the nozzle stayed at the same position for too long. As the printing speed increases, the time of nozzle staying at the same position was reduced. The EGaIn on the substrate became thinner and sometimes the printing track was interrupted. Thus, the printing speed was as 1 mm/s, and the nozzle height was chosen as 0.05 mm. The above printing parameters and vibration parameters were applied to print different EGaIn tracks, as shown in Fig. 8c–e. The printing effect is good, which further verifies the effectiveness of the above analysis.

## Conclusion

A micro-vibration-driven 3D printing method of EGaIn was proposed. With this method, the nozzle tip pierces the oxide skin of extruded EGaIn droplets, improving the time for EGaIn droplets to maintain high moldability at the nozzle tip, avoiding the generation of random large droplets in the printing process. Specific conclusions are as follows,

1. A micro-vibration driven 3D printing system for EGaIn was built. The vibration of piezoelectric ceramic was transmitted on the syringe by a compliant mechanism, and the extrusion process of EGaIn droplets under micro-vibration action was obtained by a high-speed camera.
2. Experiment results show that with the increase of vibration frequency and amplitude, the surface tension of the extrusion droplets decreases, the moldability increases, and the retention time of the droplets on the nozzle decreases. Combining the moldability, surface tension and droplet residence time, 80 V input voltage amplitude and 400 Hz vibration frequency were selected as vibration parameters. Comparing the droplet properties under vibration driving and pneumatic driving, the surface tension of EGaIn droplets under micro-vibration is small, the moldability is high, and the retention time of liquid droplets is long. All these are beneficial to control the EGaIn during extrusion.
3. The effects of printing speed and nozzle height on printing results were studied through the EGaIn 3D printing experiment. Good printing results were achieved when the printing speed was 1 mm/s and the nozzle height was 0.05 mm. The flexible elbow sensor was fabricated by micro-vibration EGaIn 3D printing on silica gel, which verified the feasibility of this method in the fabrication of flexible sensor.

The micro-vibration assisted 3D printing method for EGaIn extrusion solved the problem of random droplets in the printing structure. At the same time, the method is not limited by the printing process, materials or liquid metal formed structures. It provides a new idea for 3D printing of liquid metal.

## Data Availability

The datasets used and/or analyzed during the current study are available from the corresponding author upon reasonable request.
